# Supplementary material for: Evidence for Pentapeptide-Dependent and Independent CheB Methylesterases
Source: Int J Mol Sci. 2020 Nov 11;21(22):8459. doi: 10.3390/ijms21228459 (PMC7698151; doi:10.3390/ijms21228459)
Supplement: Supplementary file 1 [file ijms-21-08459-s001.pdf]

Supplementary Material

to

Evidence for pentapeptide dependent and independent CheB methylesterases

by

Félix Velando, José A. Gavira, Miriam Rico-Jiménez, Miguel A. Matilla and Tino Krell

```
                     10        20        30        40        50        60
                      |         |         |         |         |         |
E. coli      MSKIRVLSVDDSALMRQIMTEIINSHSDMEMVATAPDPLVARDLIKKFNPDVLTLDVEMP
S. enterica  MSKIRVLSVDDSALMRQIMTEIINSHSDMEMVATAPDPLVARDLIKKFNPDVLTLDVEMP

                     70        80        90       100       110       120
                      |         |         |         |         |         |
E. coli      RMDGLDFLEKLMRLRPMPVVMVSSLTGKGSEVTLRALELGAIDFVTKPQLGIREGMLAYN
S. enterica  RMDGLDFLEKLMRLRPMPVVMVSSLTGKGSEVTLRALELGAIDFVTKPQLGIREGMLAYS

                    130       140       150       160       170       180
                      |         |         |         |         |         |
E. coli      EMIAEKVRTAAKASLAAHKPLSAPTTLKAGPLLSSEKLIAIGASTGGTEAIRHVLQPLPL
S. enterica  EMIAEKVRTAARARIAAHKPMAAPTTLKAGPLLSSEKLIAIGASTGGTEAIRHVLQPLPL

                    190       200       210       220       230       240
                      |         |         |         |         |         |
E. coli      SSPALLITQHMPPGFTRSFADRLNKLCQIGVKEAEDGERVLPGHAYIAPGDRHMELSRSG
S. enterica  SSPAVIITQHMPPGFTRSFAERLNKLCQISVKEAEDGERVLPGHAYIAPGDKHMELARSG

                    250       260       270       280       290       300
                      |         |         |         |         |         |
E. coli      ANYQIKIHDGPAVNRHRPSVDVLFHSVAKQAGRNAVGVILTGMGNDGAAGMLAMRQAGAW
S. enterica  ANYQIKIHDGPPVNRHRPSVDVLFHSVAKHAGRNAVGVILTGMGNDGAAGMLAMYQAGAW

                    310       320       330       340
                      |         |         |         |
E. coli      TLAQNEASCVVFGMPREAINMGGVCEVVDLSQVSQQMLAKISAGQAIRI
S. enterica  TIAQNEASCVVFGMPREAINMGGVSEVVDLSQVSQQMLAKISAGQAIRI
```

Figure S1. Sequence alignment of CheB from *E. coli* K-12 substrain MG1655 and *S. enterica* serovar Typhimurium str. LT-2. Protein accession numbers are NP_416397.1 and NP_460874.1, respectively. The alignment was made using the CLUSTALW algorithm of the NPS@ suite [1] using the GONNET weight matrix, a gap opening penalty of 10 and a gap extension penalty of 0.2. Red: identical; green: highly similar; blue: weakly similar; black: dissimilar.

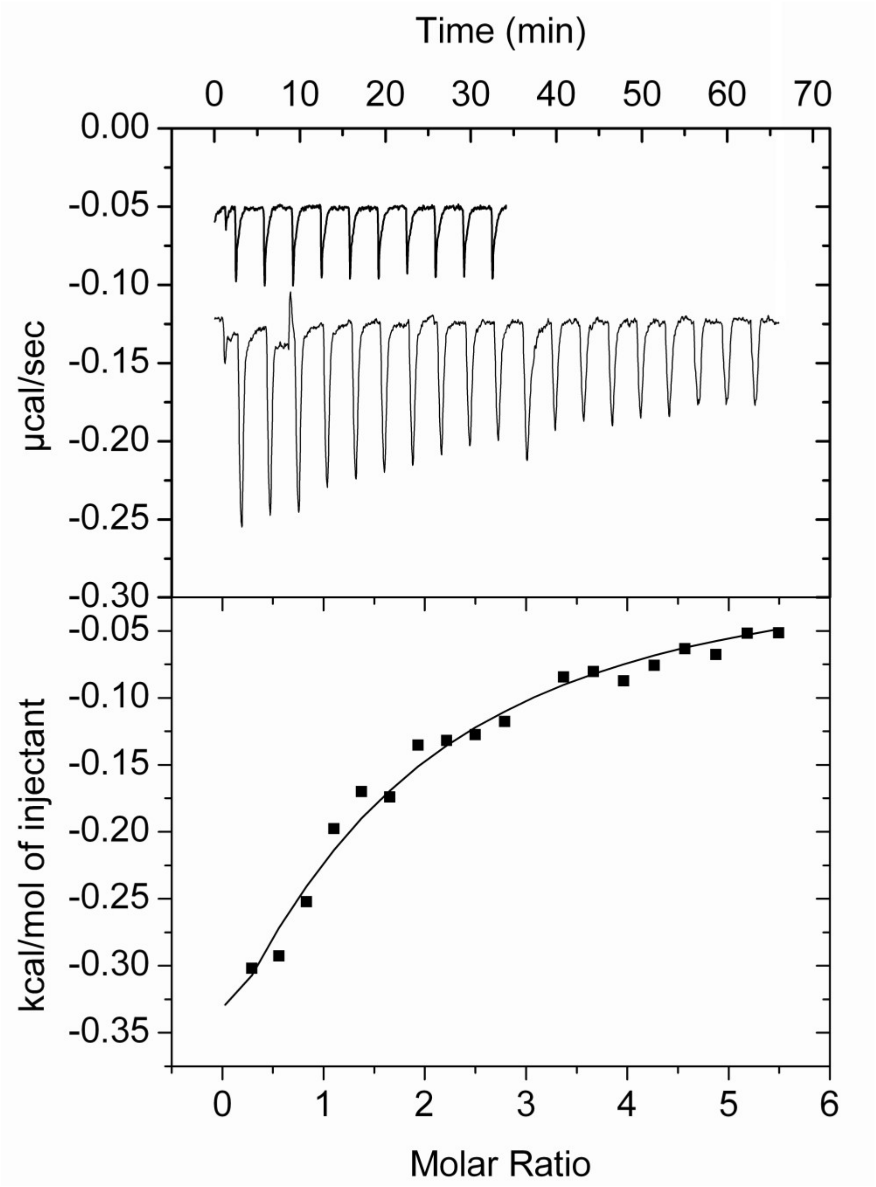

Figure S2. Microcalorimetric binding studies of the CheB$_2$ D55E mutant with the McpB-derived pentapeptide GWEEF. Upper panel: Raw data for the titration of buffer (upper curve) and the CheB$_2$ D55E mutant (38 µM) with 14 µl aliquots of the GWEEF pentapeptide (1 mM) at 25 ºC. The lower panel shows the integrated, dilution heat-corrected and concentration-normalized peak areas of the protein titration data. Data were fitted using the 'One binding site model' of the MicroCal version of ORIGIN.

```
ECA0183      LSYGNGKTA-SYASAPTRTPTL-S-LAP--AAAKNQSNND--
ECA1774      ---GASYKS-AALNRKTETPAL-A-APKNNRAEKTSAKGELA
ECA4120      -GTPAARPA-P-MAKKAQTAR----LALAPVG----NTQD--
ECA2712      AAEAPRRPQ-QRLAEKAPAAQK-P-MLLAAAGGKKGNAND--
ECA4332      ---GGSSQ---RIAPPLKRPSS-AKFSLANPKGSAGSNNQ--
ECA4333      --ESGSSQ---RTTPELKRPSS-AKLSLASPKGRTKSDSQ--
ECA4334      ---GIQTKA-PRLTSQVKQPAA-PRLALASKSGHTSSD----
ECA4335      ---GTQSQ---RAVPQVTTLSR-PKLALAGNSSNT-------
ECA3642      ---GNGHQI-ARTPAAAASLTLRPALAAPGKSGISAGEG---
ECA3902      ---GIVQQVRSSLPKSAPQPRLAPAMAIAGSS--KGNSNQ--
ECA3245      --QIASSSLIPALASVPSGLSA-PRLASAKNKNALAQDEA--
ECA1691      EDTGSFRR--TTQATAGQKPVLLAPSVNGGKKAKEGSSTD--
ECA1281      --QAVAQEH--RAASASSLAAL-PKSLLPKPTS-AGSSNA--
ECA1683      SHLSSGHSA-PARPNALAAKGR-SSLALPRQAN---TENG--
ECA2580      ---SQSDN---RVASRASSSI--PRHTLPKSVSAKAASSES-
ECA1332      ----DTQS---ALQVAAKPVRK-AQAIAPRAGKALPTSSD--
ECA1509      -SDSDQQTAFSRPAIAAPVHRAVAQSTTPLLS-VHGRHGE--
ECA2579      --ENEGRK--PKANISGLPPQ--QKYLPPAAK---QTQDS--
ECA0080      -DKDVARLQ--GSNTGNPNSGNKATARLPTLAS-RDNGND--
```

Figure S3. Alignment of the linker sequences of the 19 chemoreceptors from *Pectobacterium atrosepticum* containing terminal pentapeptides. The alignment was done using the CLUSTALW algorithm of the npsa suite [1] using the GONNET weight matrix, a gap opening penalty of 10 and a gap extension penalty of 0.2. No significant sequence conservation is observed.

ECA0080 ANSLQEQASKLATLMSVFRISDKDVARLQGSNTGNPNSGNKATARLPTLASRDNGNDNWTTF
DSC hhhhhhhhhhhhhhhhhhhhcchhhhhhcccccccccccchhhhhhhhhhcccccccccccc
MLRC hhhhhhhhhhhhhhhhhhhccccceeeecccccccccccccccccccccccccccccccccc
PHD hhhhhhhhhhhhhheeeeeecccccccccccccccccccccccccccccccccccccccccc
Sec.Cons. hhhhhhhhhhhhhhhhhhh?cccc????cccccccccccccccccccccccccccccccccc

ECA0183 SAALSLQAQASVLAEAVSTFKLLSYGNGKTASYASAPTRTPTLSLAPAAAKNQSNNDNWTTF
DSC hhhhhhhhhhhhhhhhhhhhhhhccccccccccccccccccccccccchhcccccccccccc
MLRC hhhhhhhhhhhhhhhhhhhhhecccccccccccccccccccccccccccccccccccccccc
PHD hhhhhhhhhhhhhhhhhhhheeccccccccccccccccccccccccchhhhhcccccccccc
Sec.Cons. hhhhhhhhhhhhhhhhhhhhhecccccccccccccccccccccccccchhcccccccccccc

ECA1281 AAAALEEQARFLQNAVEVFKINQAVAQEHRAASASSLAALPKSLLPKPTSAGSSNANWETF
DSC hhhhhhhhhhhhhhhhhhhhhhhhhhhhhhhhhhhhhhhhhhccccccccccccccccccc
MLRC hhhhhhhhhhhhhhhhhhhhhhhhhhhhhhhhhhhhhhhcccccccccccccccccccccc
PHD hhhhhhhhhhhhhhhhheeechhhhhhhhhccccccccccccccccccccccccccccccc
Sec.Cons. hhhhhhhhhhhhhhhhhhhhhhhhhhhhhhhhhhhhhhhcccccccccccccccccccccc

ECA1332 SSAALSLEEQAALLNQTVSLFQLSDTQSALQVAAKPVRKAQAIAPRAGKALPTSSDNWEKF
DSC hhhhhhhhhhhhhhhhhhhhhhhcccchhhhhccchhhhhhhcchhhhhhccccccccccc
MLRC hhhhhhhhhhhhhhhhhhhhhccccchhhhhhccchhhhhhhccccccccccccccccccc
PHD hhhhhhhhhhhhhhhhhheeeeecccccccccccccccccccccccccccccccccccccc
Sec.Cons. hhhhhhhhhhhhhhhhhhhhh??cccchhhhhccchhhhhhhccccccccccccccccccc

ECA1509 LDEQTSELSAVVDVFNLDSDSDQQTAFSRPAIAAPVHRAVAQSTTPLLSVHGRHGEGWEKF
DSC hhhhhhhhhhhhheeeccccccccccccccccccccccccccccccccccccccccccccc
MLRC hhhhhhhhhhhhhhecccccccccccchcccchhhhhhhhhhccccceeecccccccccccc
PHD hhhhhhhhhhheeeeeecccccccccccccccccccccccccccccccccccccccccccc
Sec.Cons. hhhhhhhhhhhhheeeccccccccccccccccccccccccccccccccccccccccccccc

ECA1683 AASLEEQAALLTRAVATFKLSSHLSSGHSAPARPNALAAKGRSSLALPRQANTENGNWETF
DSC hhhhhhhhhhhhhhhhhhhhccccccccccccchhhhhhcccchhhhhhhhcccccccccc
MLRC hhhhhhhhhhhhhhhhhhhcccccccccccccccccccccccccccccccccccccccccc
PHD hhhhhhhhhhhhhhheeeeeccccccccccccccccccccccccccccccccccccccccc
Sec.Cons. hhhhhhhhhhhhhhhhhhh?ccccccccccccccccccccccccccccccccccccccccc

ECA1691 LEEQVKVLNQAVAVFRLSEDTGSFRRTTQATAGQKPVLLAPSVNGGKKAKEGSSTDNWETF
DSC hhhhhhhhhhhhhhhhhcccccchhhhhhhhhcccceeeeccccccccccccccccccccc
MLRC hhhhhhhhhhhhhhhhccccccccchcccccccccceeecccccccccccccccccccccc
PHD hhhhhhhhhhhhheeeecccccccccccccccccccccccccccccccccccccccccccc
Sec.Cons. hhhhhhhhhhhhhhhh?cccccccchcccccccccceeecccccccccccccccccccccc

ECA1774 ALSLQDQANSLARTVSVFNLGASYKSAALNRKTETPALAAPKNNRAEKTSAKGELADWTTF
DSC hhhhhhhhhhhhhhhhhhhhccchhhhhccccccccccccccccccccccccccccccccc
MLRC hhhhhhhhhhhhhhhhhhcccccccccchccccccccccccccccccccccccchchhccc
PHD hhhhhhhhhhhhhhhhheeeccccccccccccccccccccccccccccccccccccccccc
Sec.Cons. hhhhhhhhhhhhhhhhhh??ccccccccccccccccccccccccccccccccccccccccc

ECA2579 SSISANSLEEQSAILESMVANFRLSENEGRKPKANISGLPPQQKYLPPAAKQTQDSGWTTF
DSC hhhhhhhhhhhhhhhhhhhhhhhhcccccchhhhhhhccccccchhccccccccccccccc
MLRC hhhhhhhhhhhhhhhhhhhhhhhhccccccccccccccccccccccccccccccccccccc
PHD hhhhhhhhhhhhhhhhhhhhheeeccccccccccccccccccccccccccccccccccccc
Sec.Cons. hhhhhhhhhhhhhhhhhhhhhhhhccccccccccccccccccccccccccccccccccccc

ECA2580 SISANSLEEQAVLLEKLLEHFRVSQSDNRVASRASSSIPRHTLPKSVSAKAASSESDWTSF
DSC hhhhhhhhhhhhhhhhhhhhhhhccccccccccccccccccccchhhhhhhhccccccccc
MLRC hhhhhhhhhhhhhhhhhhhhhccccccccccccccccccccccccccchhccccccccccc
PHD hhhhhhhhhhhhhhhhhhhheeeeccccccccccccccccccccccccccccccccccccc
Sec.Cons. hhhhhhhhhhhhhhhhhhhhh??ccccccccccccccccccccccccchhccccccccccc

ECA2712 ASLEDQARQLTEAVSVFQLSAAEAPRRPQQRLAEKAPAAQKPMLLAAAGGKKGNANDNWETF

```
DSC         hhhhhhhhhhhhhhhhhhhhhhhhccchhhhhhhhhhhhhhhhhhhcchhcccccccccccc
MLRC        hhhhhhhhhhhhhhhhhhhccccccccchhhhhhhcccccccეeeecccccccccccccccc
PHD         hhhhhhhhhhhhhhheeeeccccccccchhhhhhhhccccchhhhccccccccccccccccc
Sec.Cons.   hhhhhhhhhhhhhhhhhhhccccccccchhhhhhhhccccchhhh?cccccccccccccccc

ECA3245     QSLQEQASTLSQLVGQFIVGQIASSSLIPALASVPSGLSAPRLASAKNKNALAQDEAGWQRF
DSC         hhhhhhhhhhhhhhhhheecccccccccccccccccccccchhhhhhhhhhhhhhhhhhhccc
MLRC        hhhhhhhhhhhhhhhhhhhccccchhhhhhhhccccccccchhhhhhhhhhhhhhhcccccc
PHD         hhhhhhhhhhhhhhhhheecccccccccccccccccccccccchhhhhhhhhhhhhhcccccc
Sec.Cons.   hhhhhhhhhhhhhhhhheeccccccccccccccccccccccchhhhhhhhhhhhhhhcccccc

ECA3642     AATQSLQQQAAQLAETVSQFRLGNGHQIARTPAAAASLTLRPALAAPGKSGISAGEGDWTSF
DSC         hhhhhhhhhhhhhhhhhhhhhhhccccccccccccccchhhhhhcccccccccccccccccc
MLRC        hhhhhhhhhhhhhhhhhhhhhcccccccccccccchhhhhhhhccccccccccccccccccc
PHD         hhhhhhhhhhhhhhhhheeeeecccccccccccccccccccccccccccccccccccccccc
Sec.Cons.   hhhhhhhhhhhhhhhhhhhhh?cccccccccccccccchhhhhccccccccccccccccccc

ECA3902     AAVSLEEQAARLTQAVAVFKLSGIVQQVRSSLPKSAPQPRLAPAMAIAGSSKGNSNQNWETF
DSC         hhhhhhhhhhhhhhhhhheecccccccccccccccccccccccchhcccccccccccccccc
MLRC        hhhhhhhhhhhhhhhhhhhhhcchhhhhhhccccccccccccccccccccccccccccccccc
PHD         hhhhhhhhhhhhhhceeeeeeccccccccccccccccccccccccccccccccccccccccc
Sec.Cons.   hhhhhhhhhhhhhhhhhhee?ccccccccccccccccccccccccccccccccccccccccc

ECA4120     VEESANAATSLREQSSHLVTLMNHFHLRGTPAARPAPMAKKAQTARLALAPVGNTQDNWEKF
DSC         hhhhhhhhhhhhhhhhhhhhhhheeeecccccccchhhhhhhhhhhheecccccccccccccc
MLRC        hhhhhhhhhhhhhhhhhhhhhhhhhhccccccccccchhhhhhhhheeeccccccccccccc
PHD         hhhhhhhhhhhhhhhhhhhhhhhheeecccccccccchhhccchhhhhhhhhcccccccccccc
Sec.Cons.   hhhhhhhhhhhhhhhhhhhhhhhheeeccccccccchhhhhhhhhhee?ccccccccccccc

ECA4332     ASAADSLSDQAAHLVELMKVFIVEGGSSQRIAPPLKRPSSAKFSLANPKGSAGSNNQNWEQF
DSC         hhhhhhhhhhhhhhhhhhhhhhhhcccccccccccccccccccccccccccccccccccccc
MLRC        hhhhhhhhhhhhhhhhhhhhheeccccccccccccccccccccecccccccccccccccccc
PHD         hhhhhhhhhhhhhhhhhhheeeeecccccccccccccccccccccccccccccccccccccc
Sec.Cons.   hhhhhhhhhhhhhhhhhhhhhee?cccccccccccccccccccccccccccccccccccccc

ECA4333     TSAADSLSDQAAHLVELMKVFIVESGSSQRTTPELKRPSSAKLSLASPKGRTKSDSQNWETF
DSC         hhhhhhhhhhhhhhhhhhhhhhhccccccccccccccccccccccccccccccccccccccc
MLRC        hhhhhhhhhhhhhhhhhhhhheecccccccccccccccccceeccccccccccccccccccc
PHD         hhhhhhhhhhhhhhhhhheeeeeecccccccccccccccccccccccccccccccccccccc
Sec.Cons.   hhhhhhhhhhhhhhhhhhhhheeccccccccccccccccccccccccccccccccccccccc

ECA4334     ASAADSLSQQARTLLELMGVFKLNGIQTKAPRLTSQVKQPAAPRLALASKSGHTSSDNWETF
DSC         hhhhhhhhhhhhhhhhhhhhhhhcccchhhhhhhhccccccccccccccccccccccccccc
MLRC        hhhhhhhhhhhhhhhhhhhhhhccccccccchhhhccccccccccceccccccccccccccc
PHD         hhhhhhhhhhhhhhhhheeeeeeccccccccccccccccccccccccccccccccccccccc
Sec.Cons.   hhhhhhhhhhhhhhhhhhhhhh?cccccccchhhhccccccccccccccccccccccccccc

ECA4335     LVEESASAADSLSQQARNLVELMGVFKIDGTQSQRAVPQVTTLSRPKLALAGNSSNTNWETF
DSC         hhhhhhhhhhhhhhhhhhhhhhhhhhhccccccccccccccccccccccccccccccccccc
MLRC        hhhhhhhhhhhhhhhhhhhhhhhhehecccccccccccccccccccceeecccccccccccc
PHD         hhhhhhhhhhhhhhhhhhhhhheeeeeecccccccccccccccccccccccccccccccccc
Sec.Cons.   hhhhhhhhhhhhhhhhhhhhhhhhehecccccccccccccccccccccccccccccccccccc
```

Figure S4. Secondary structure prediction of the C-terminal segment of pentapeptide containing chemoreceptors of *P. atrosepticum*. The appendices harboring linker and pentapeptide are shaded in yellow. h: α-helix, e: β-strand, c: coil. Sec. Cons: Secondary structure consensus derived from the DSC, MLRC and PHD algorithms. This analysis was carried out using the npsa server (https://npsa-prabi.ibcp.fr/cgi-bin/npsa_automat.pl?page=/NPSA/npsa_seccons.html) [1].

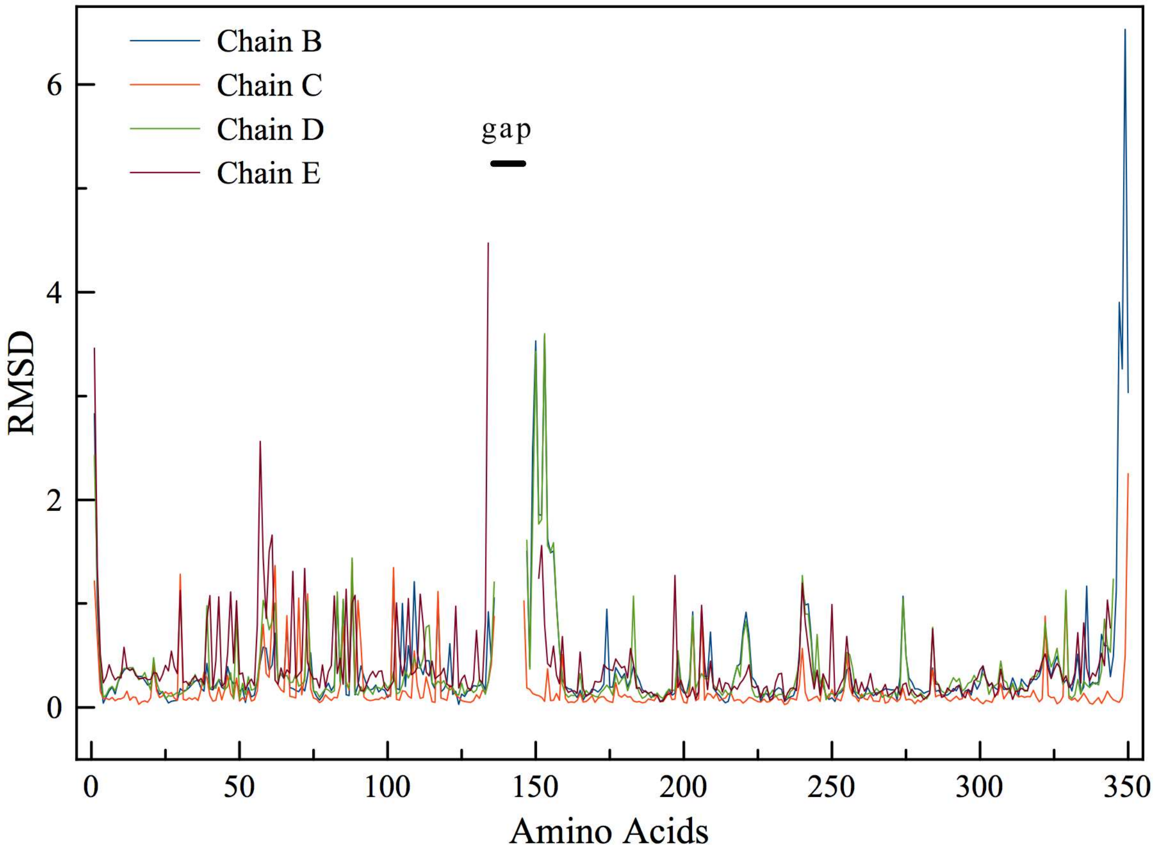

Figure S5. Analysis of the structure of *P. atrosepticum* CheB. Structural superimposition of chain A onto the remaining 4 chains of the asymmetric unit. Shown are rmsd of Cα atoms.

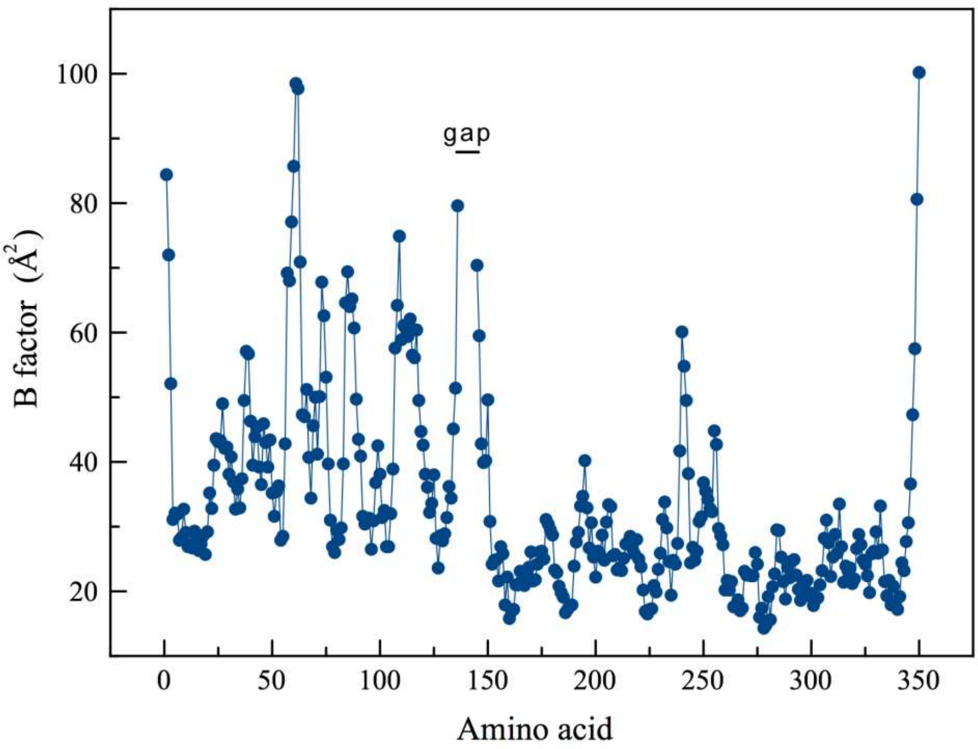

Figure S6. B-factors of Cα atoms of *P. atrosepticum* chain B. The gap caused by the low electron density in the crystal structure of CheB_Pec is indicated.

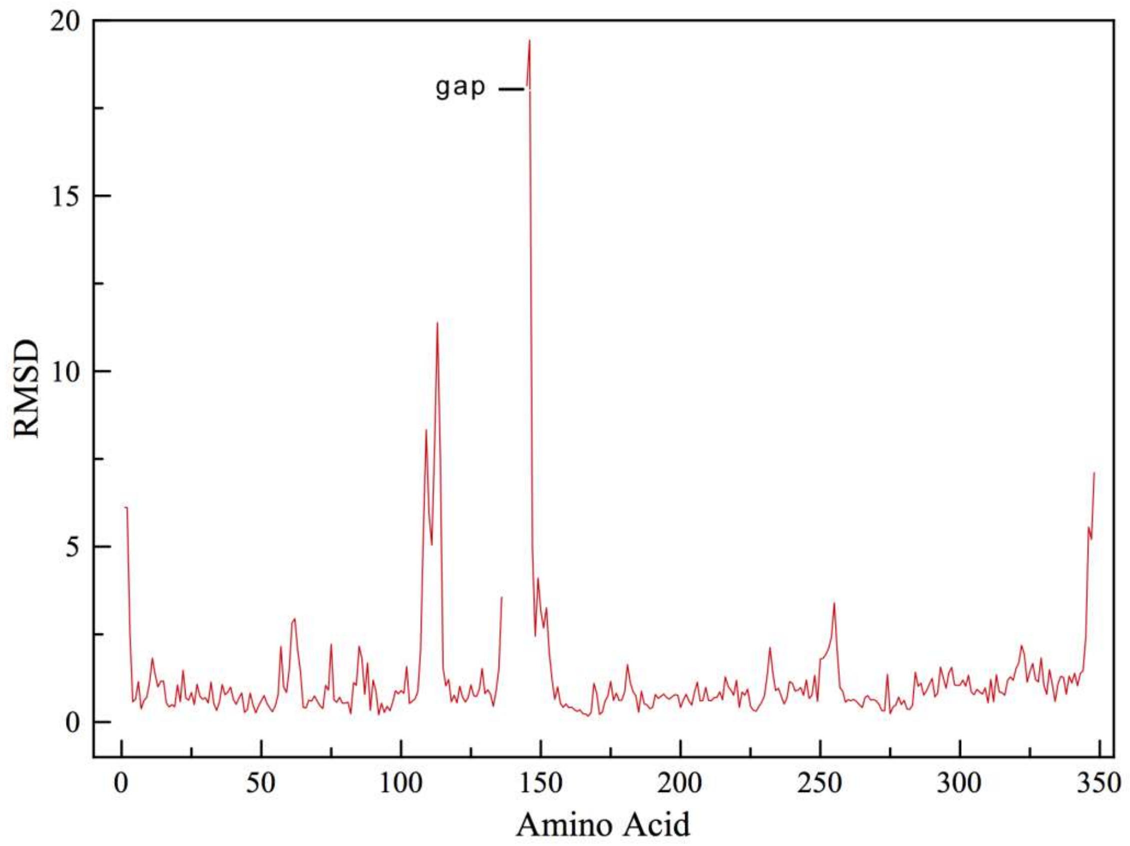

Figure S7. Structural superimposition of chain A of *P. atrosepticum* CheB with CheB of *S. enterica* sv. Typhimurium (pdb ID 1A2O). Shown are rmsd of Cα atoms.

Table S1. *P. atrosepticum* SCRI1043 chemoreceptors with a C-terminal pentapeptide. Shown are the pentapeptide sequence, the chemoreceptor locus tag and the LBD type of the receptor.

**Pentapeptide sequence**	**receptor**	**LBD type**
GWQRF	ECA3245	4HB[1]
GWEKF	ECA1509	4HB
GWTTF	ECA2579	HBM
DWTSF	ECA3642	4HB
	ECA2580	HBM
DWTTF	ECA1774	4HB
NWTTF	ECA0183	4HB
	ECA0080	HBM
NWEKF	ECA4120	4HB
	ECA1332	4HB
NWEQF	ECA4332	4HB
NWETF	ECA4333	4HB
	ECA4334	4HB
	ECA4335	4HB
	ECA1281	4HB
	ECA1691	4HB
	ECA1683	4HB
	ECA3902	4HB
	ECA2712	4HB

[1]4HB, four helix bundle domain; HBM, helical bimodular domain

Table S2. Structural alignment of chains A to E of *P. atrosepticum* CheB amongst each other and with related structures. Data were calculated using MATRAS [2]. Pdb ID 1a2o: CheB from *S. enterica* sv. Typhimurium; pdb ID 1chD: methylesterase domain of *S. enterica* sv. Typhimurium CheB methylesterase; pdb ID 3sft: methylesterase domain of *Thermotoga maritima* CheB; pdb ID 3t8y: REC domain of *T. maritima* CheB.

	Cα rmsd (Å)								
	A	B	C	D	E	1a2o	1chd	3sft	3t8y
Aligned amino acids						347	198	198	138
CheB (*P. atrosepticum*) chain A	-	0.634	0.234	0.593	0.517	1.420	0.949	1.237	2.083
CheB (*P. atrosepticum*) chain B	0.634	-	0.607	0.252	0.522	1.468	0.878	1.163	2.775
CheB (*P. atrosepticum*) chain C	0.234	0.607	-	0.573	0.521	1.390	0.937	1.235	2.805
CheB (*P. atrosepticum*) chain D	0.593	0.252	0.573	-	0.527	1.397	0.896	1.168	2.758
CheB (*P. atrosepticum*) chain E	0.517	0.522	0.521	0.527	-	1.207	0.788	1.211	2.821
CheB (*S. enter*) pdb 1a2O	1.420	1.468	1.390	1.397	1.207	-	1.139	1.209	2.357
CheB cat. domain (*S. enter*) pdb 1chd	0.949	0.878	0.937	0.896	0.788	1.139	-	1.058	4.782
CheB cat. domain (*T. mari*) pdb 3sft	1.237	1.163	1.235	1.168	1.211	1.209	1.058	-	4.685
CheB REC domain (*T. mari*) pdb 3t8y	2.083	2.775	2.805	2.758	2.821	2.357	4.782	4.685	-

Table S3. Oligonucleotides used in this study.

#	name	sequence (5'-3')	purpose
1	pET28_CheB$_1$_f	GTCGCCCTCTGAGGAGTACATATGGCAGTC	Construction of pET28b-CheB$_1$
2	pET28_CheB$_1$_r	CATTCCTGCACGGATCCTTACTGGCACGCCTC	
3	pET28_CheB$_2$_f	GGAGAGCCCATATGCCCATCAGTGTCCT	Construction of pET28b-CheB$_2$
4	pET28_CheB$_2$_r	CCAGGATCCCGCTCAGAGG	
5	pET28_CheB$_3$_f	CTGATCGGAGAAGCACACATATGAGGATCGGA	Construction of pET28b-CheB$_3$
6	pET28_CheB$_3$_r	GGAGTGGATCCCGGCTAATCGAATAC	
7	pET28_CheB$_4$_f	CCTGGTCGGCCCATATGAGTGAG	Construction of pET28b-CheB$_4$
8	pET28_CheB$_4$_r	TCGGATCCTGGTTCATGTTTCGACTC	
9	pET28_CheB$_2$_ D55E_f	GACGTGATCAGCCTCGAAGTGGAAATGCCGCGAATGGAC	Construction of pET28b-CheB$_2$ D55E
10	pET28_CheB$_2$_ D55E_r	GGCATTTCCACTTCGAGGCTGATCACGTCCGGCGCGTGC	
11	pET28_CheB_Pec_f	TAATCATATGAAAAGTACACCATCGCAAAATC	Construction of pET28b-CheB_Pec
12	pET28_CheB_Pec_r	TAATGGATCCCTCTTATCTTGCTCATTATCTTTCCTTAG	
13	pUC18Not_cheB_up_f	TAATGCATGCCTATTCTGACGCAGATGGTTGAC	Construction of pUC18Not_ΔcheB
14	pUC18Not_cheB_up_r	CAACATTCGCTGGCTCACGGATCCTAGGGCAGAATCATCAACGCA	
15	pUC18Not_cheB_down_f	GCGTTGATGATTCTGCCCGGATCCCAGGTGAGCCAGCGAATGTTG	Construction of pUC18Not_ΔcheB
16	pUC18Not_cheB_down_r	TAATCATATGAGCCTCTGCTGCTTCTGCTA	
17	pUC18Not_cheA_up_f	TAATGAATTCGCGAGATATCCTGCGTGCG	Construction of pUC18Not_ΔcheA
18	pUC18Not_cheA_up_r	CTCCGATGGATCCAACAATAATAAGTGTTG	
19	pUC18Not_cheA_down_f	TAATGGATCCGACCTGTATCCGTTAGCTGGCG	Construction of pUC18Not_ΔcheA
20	pUC18Not_cheA_down_r	TAATAAGCTTCAATACGCTATCGACCAGCGC	

Table S4. Data collection and refinement statistics of the 3D structure of *P. atrosepticum* CheB. Values in parentheses are for the highest-resolution shell.

Protein	CheB
**Pdb identifier**	6YMZ
**Data collection**	
Beamline	Xaloc (ALBA)
Space Group	I 4
Cell dimensions a=b, c (Å)	148.74, 206.34
Protein chains in ASU	5
Resolution (Å)	62.43 - 2.30 (2.38 - 2.30)
R_{merge} (%)	78.78 (180)
I/σ_I	40.01 (3.41)
Completeness (%)	98.12 (98.06)
Unique reflections	98138 (9778)
Multiplicity	26.2 (25.4)
CC(1/2)	0.83 (0.65)
**Refinement**	
Resolution (Å)	2.30
R_{work}/R_{free} (%)	19.18/22.62
No. of atoms	13992
Protein	13433
Ligands	65
Water	494
B-factor (Å^2)	43.92
R.m.s deviations	
Bond lengths (Å)	0.01
Bond angles (^{0})	1.15
Ramachandran (%)	
Favoured	98.44
Allowed	1.38
Outliers	0.18

References

1. Combet, C.; Blanchet, C.; Geourjon, C.; Deleage, G., NPS@: network protein sequence analysis. *Trends Biochem. Sci.* **2000,** 25, 147-50.
2. Kawabata, T., MATRAS: A program for protein 3D structure comparison. *Nucleic Acids Res.* **2003,** 31, 3367-9.
